# ﻿The South American moth *Rheumapteramochica* (Dognin, 1904) (Lepidoptera, Geometridae, Larentiinae) rediscovered after more than a century of anonymity

**DOI:** 10.3897/zookeys.1085.76868

**Published:** 2022-02-07

**Authors:** Héctor A. Vargas, M. Alma Solis, Marcelo Vargas-Ortiz

**Affiliations:** 1 Departamento de Recursos Ambientales, Facultad de Ciencias Agronómicas, Universidad de Tarapacá, Arica, Chile Universidad Tarapacá Arica Chile; 2 Systematic Entomology Laboratory, PSI, Agricultural Research Service, U.S. Department of Agriculture, c/o National Museum of Natural History, Smithsonian Institution, P.O. Box 37012, MRC 168, DC Washington, USA National Museum of Natural History, Smithsonian Institution Washington United States of America; 3 Programa de Doctorado en Sistemática y Biodiversidad, Departamento de Zoología, Facultad de Ciencias Naturales y Oceanográficas, Universidad de Concepción, Concepción 4030000, Chile Universidad de Concepción Concepción Chile

**Keywords:** DNA barcodes, Fabaceae, genitalia, Rheumapterini, *Sennabirostris*

## Abstract

*Rheumapteramochica* (Dognin, 1904) (Lepidoptera, Geometridae, Larentiinae) is reported from Chile for the first time. It was described from the western slopes of the Andes of southern Peru more than 100 years ago, and was recently rediscovered in Chile after larvae were collected and reared on the shrub Sennabirostrisvar.arequipensis (Meyen ex Vogel) H.S. Irwin & Barneby (Fabaceae). This discovery expands the known distribution of this moth and provides its first host plant record. The genitalia of *R.mochica* are described and illustrated for the first time and compared to those of *R.affirmata* (Guenée, [1858]). A maximum likelihood analysis based on mitochondrial DNA sequences clustered *R.mochica* as sister to *R.affirmata* with 3.6–3.8% divergence (K2P). A lectotype is designated for *Calocalpemochica* Dognin, 1904.

## ﻿Introduction

*Rheumaptera* Hübner, 1822 (Lepidoptera, Geometridae, Larentiinae) is a widespread moth genus with 66 species, mostly from the Palearctic and Oriental regions; 14 species are recorded in the Western Hemisphere (Parsons et al. 1999). A recent molecular phylogenetic analysis strongly supports its monophyly and resulted in the transfer of three New World species from *Coryphista* Hulst, 1896 and *Triphosa* Stephens, 1829 to *Rheumaptera* (Brehm et al. 2019).

The Neotropical *Rheumapteramochica* (Dognin, 1904) was originally described in *Calocalpe* Hübner, [1825], a junior synonym of *Rheumaptera* (Parsons et al. 1999). The species was based on two syntypes, a male and a female, from Arequipa on the western slopes of the Andes in southern Peru (Dognin 1904). No additional specimens have been reported in the literature since its original description. However, recently, adults of *R.mochica* were reared from larvae collected on a native shrub in northern Chile, a discovery that sheds light on this obscure geometrid moth.

The goals of this study were to confirm the identity of the reared adults, describe and illustrate their genitalia, and analyze their DNA from the COI barcode region (sensu Hebert et al. 2003) for the first time. Also, we report the host plant of *R.mochica* for the first time and expand its known distribution range. We designate a lectotype for *Calocalpemochica* Dognin, 1904, to stabilize its nomenclature.

## ﻿Material and methods

### ﻿Specimens

Adults of *R.mochica* were reared from folivorous larvae collected on the native shrub Sennabirostrisvar.arequipensis (Meyen ex Vogel) H.S. Irwin & Barneby (Fabaceae), near the villages of Belén (18°28'01"S, 69°30'37"W), Chapiquiña (18°23'34"S, 69°31'55"W), and Socoroma (18°16'03"S, 69°36'01"W) in the Parinacota Province of northern Chile, at about 3200–3400 m elevation on the western slopes of the Andes. Genitalia dissections were performed using standard procedures. Images of the genitalia were captured with a Sony CyberShot DSC-HX200V digital camera attached to a Leica M125 stereomicroscope and a Micropublisher 3.3 RTV-QImaging digital camera attached to an Olympus BX51 optical microscope. The distribution map was generated using SimpleMappr (Shorthouse 2010).

### ﻿Abbreviations of institutional collections

**DZUP**Pe. Jesus de Santiago Moure Collection, Universidade Federal do Paraná, Paraná, Brazil;

**IDEA** Colección Entomológica de la Universidad de Tarapacá, Arica, Chile;

**USNM**United States National Museum of Natural History, Smithsonian Institution, Washington, D.C., USA.

### ﻿DNA extraction, sequencing, and analysis

Genomic DNA was extracted from legs of five adults from Socoroma following the procedures described in Huanca-Mamani et al. (2015). DNA purification, PCR amplification, and sequencing of the barcode fragment with the primers LCO-1490 and HCO-2198 (Folmer et al. 1994) were performed by Macrogen Inc. (Seoul, South Korea) following the PCR program described in Escobar-Suárez et al. (2017). Additional sequences (Table 1) with species-level identification and 658 base pair (bp) length were downloaded from BOLD (Ratnasingham and Hebert 2007) for analysis, including congenerics and representatives of the phylogenetically close genera *Philereme* Hübner, [1825] and *Triphosa* Stephens, 1829 as outgroups, following a recent phylogeny of Geometridae (Brehm et al. 2019). The software MEGAX (Kumar et al. 2018) was used to perform sequence alignment with the ClustalW method, to estimate sequence divergence with the Kimura 2-Parameter (K2P) method, and choose the nucleotide substitution model using the lowest Bayesian information criterion value. A substitution saturation test, Xia test (Xia et al. 2003), was performed with the software DAMBE7 (Xia 2018), to evaluate the utility of the alignment for phylogenetic inference (ISS was lower than ISS.C). The phylogenetic tree was inferred through a maximum likelihood (ML) analysis with 1000 bootstrap replications and GTR+G as an evolutionary model in the software MEGAX (Kumar et al. 2018).

**Table 1.** DNA barcode sequences used in the molecular analysis.

Species	BOLD accession	GenBank accession	Country
*Rheumapteraaffirmata* (Guenée, [1858])	GWOTG471-12		Bolivia
*Rheumapteracervinalis* (Scopoli, 1763)	GBMIN33816-13	JF784768	Finland
*Rheumapteraexacta* (Butler, 1882)	GWOR2488-08		Chile
*Rheumapterafuegata* (Staudinger, 1899)	GWOR2273-08		Chile
*Rheumapterahastata* (Linnaeus, 1758)	ALLEP184-13		Canada
*Rheumapteraincertata* (Staudinger, 1882)	GBGL30834-19	KX343620	Kyrgyzstan
*Rheumapterameadii* (Packard, 1874)	GWNR428-07	HQ647618	Canada
*Rheumapteramochica* (Dognin, 1904)	RHEMO001-22	OK484459	Chile
*Rheumapteramochica* (Dognin, 1904)	RHEMO002-22	OK484460	Chile
*Rheumapteraundulata* (Linnaeus, 1758)	BBLPB099-10	JF842111	Canada
*Phileremetransversata* (Hufnagel, 176)	CGUKB362-09		United Kingdom
*Phileremevetulata* (Denis & Schiffermüller, 1775)	CGUKB463-09		United Kingdom
*Triphosadubitata* (Linnaeus, 1758)	FGMLD158-13		Germany
*Triphosasabaudiata* (Duponchel, 1830)	GWOR4460-09	KX071922	Greece

## ﻿Results

### ﻿ Rheumaptera mochica

Taxon classificationAnimaliaLepidopteraGeometridae

(Dognin, 1904)

66BFC05A-1FEC-5E2D-B8E0-05B4CDFAB72D

Calocalpe mochica Dognin, 1904: 361.
Rheumaptera mochica : Parsons et al. 1999.

#### Type material examined.

**Figure 1.**
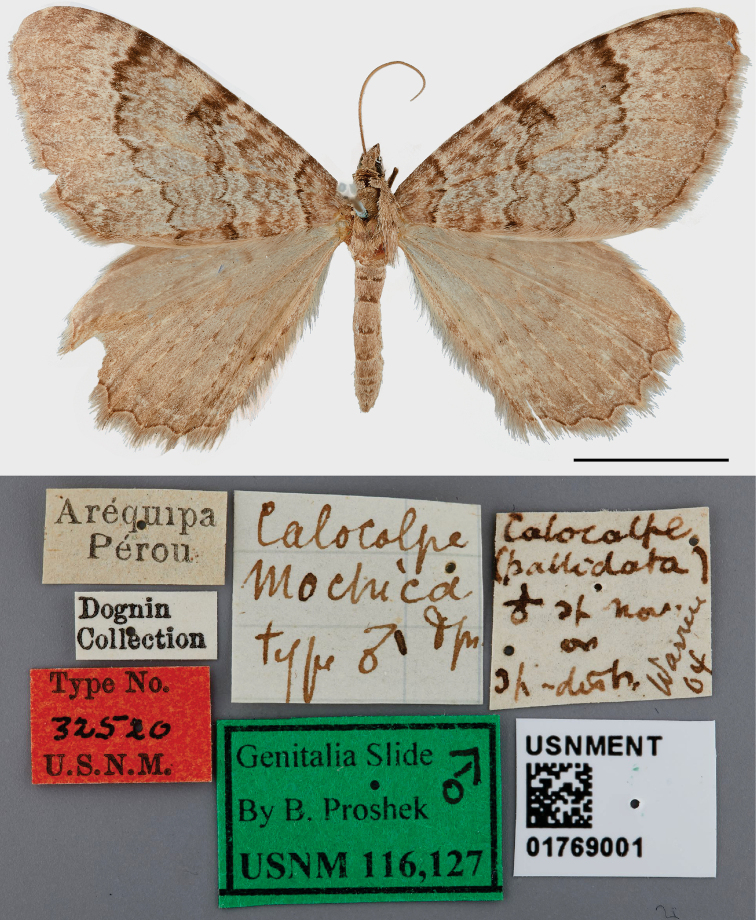
*Rheumapteramochica* (Dognin, 1904), male lectotype, dorsal view. Scale bar: 10 mm.

**Figure 2.**
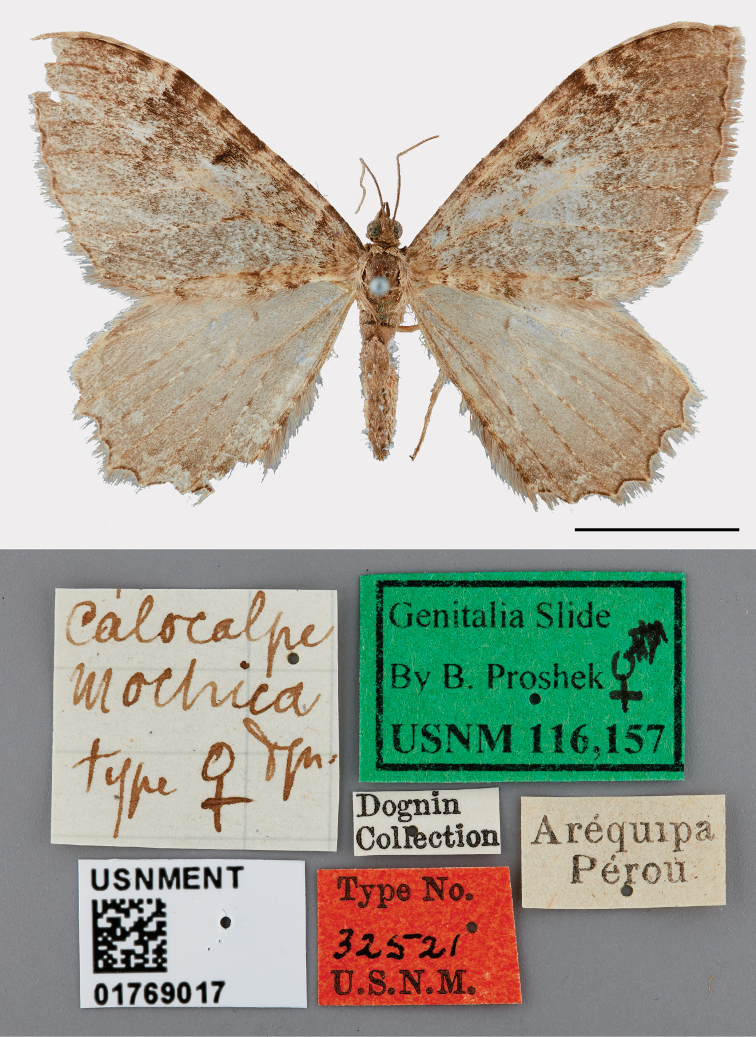
*Rheumapteramochica* (Dognin, 1904), female paralectotype, dorsal view. Scale bar: 10 mm.

Peru. The male ***lectotype*** and one female ***paralectotype*** are here designated (Figs 1, 2). The lectotype and its genitalia slide are deposited in the USNM and bear the following labels: Aréquipa/Pérou; *Calocalpe*/*mochica*/Dgn/type ♂ [Dognin handwriting]; *Calocalpe*/(*pallidata*)/Warren 04 [Dognin handwriting]; Dognin/Collection; Type No./32520/USNM [red label]; Genitalia Slide ♂/by B. Proshek/ USNM 116,127 [green label]; USNMENT/01769001. The paralectotype and its genitalia slide are deposited in the USNM and bear the following labels: Aréquipa/Pérou; *Calocalpe*/*mochica*/Dgn/type ♀ [Dognin handwriting]; Dognin/Collection; Type No./32521/USNM [red label]; USNMENT/01769017.

#### Additional material examined.

Chile – Parinacota Province • 2 ♂♂; Socoroma, 18°16'03"S, 69°36'01"W, December 2017, H.A. Vargas leg., ex larva Sennabirostrisvar.arequipensis, October 2017; [genitalia slide numbers] HAV1423, 1454; IDEA • 5 ♀♀; same data as previous; [genitalia slide numbers] HAV1424, 1440, 1455, 1456, 1457; IDEA • 2 ♂♂; same locality, August 2009, H.A. Vargas leg., ex larva Sennabirostrisvar.arequipensis, June 2009; [genitalia slide numbers] HAV1335, 1439; IDEA • 1 ♂; same locality, December 2008, H.A. Vargas leg., ex larva Sennabirostrisvar.arequipensis, October 2008; [genitalia slide number] HAV1438; IDEA • 1 ♂; Chapiquiña, 18°23'34"S, 69°31'55"W, October 2015, H.A. Vargas leg., ex larva Sennabirostrisvar.arequipensis, August 2015; [genitalia slide number] HAV1333; IDEA • 1 ♀; same data as for preceding; [genitalia slide number] HAV1339; IDEA • 1 ♂; Belén, 18°28'01"S, 69°30'37"W, October 2015, H.A. Vargas leg., ex larva Sennabirostrisvar.arequipensis, August 2015; [genitalia slide number] HAV1337; IDEA • 1 ♀; same data as previous; [genitalia slide number] HAV1334; IDEA.

#### Identification.

The identification of the Chilean specimens as *R.mochica* was based on comparisons of their male genitalia with those of the lectotype.

**Figures 3–4.**
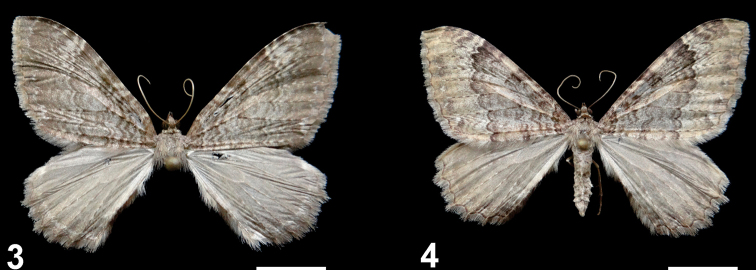
*Rheumapteramochica* (Dognin, 1904), male adults from northern Chile. Scale bar: 10 mm.

***Wing pattern*** (Figs 3, 4) The forewing pattern of the Chilean specimens of *R.mochica* is slightly variable; the area between the postmedial line and the termen can be mostly light whitish-brown or mostly dark greyish-brown. This variation is not associated to sex.

**Figure 5.**
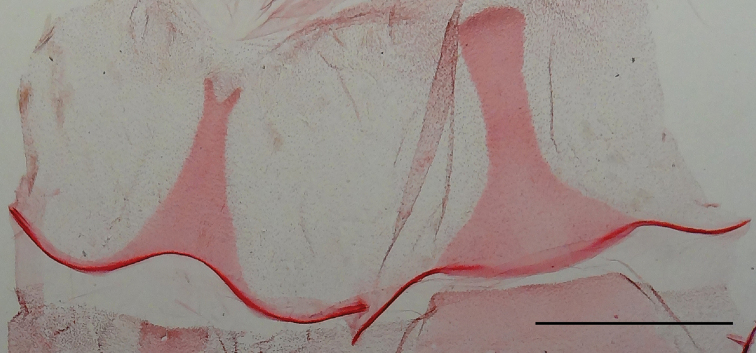
*Rheumapteramochica* (Dognin, 1904), tergum (right) and sternum (left) of male abdominal segment VIII. Scale bar: 1 mm.

***Male segment****VIII* (Fig. 5) Tergum a narrow, longitudinal plate; anterior third triangular; distal two-thirds a narrow stripe; anterior margin widely rounded, laterally projected; posterior margin rounded. Sternum a narrow triangular longitudinal plate; anterior margin widely excavated, laterally projected; posterior margin narrowly excavated.

**Figures 6–11.**
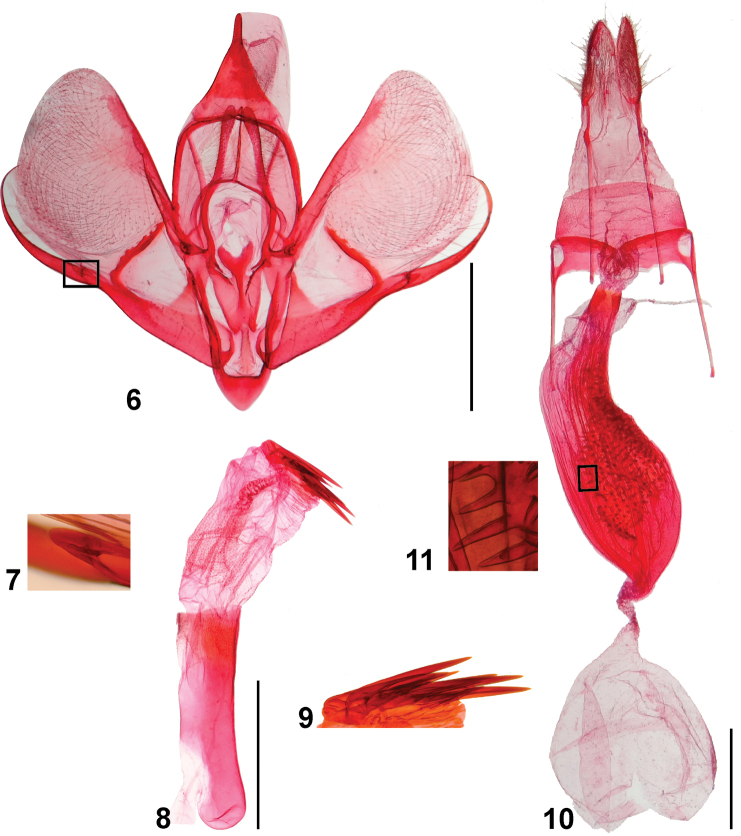
*Rheumapteramochica* (Dognin, 1904), genitalia **6** male genitalia, ventral view, phallus removed **7** basal process of sacculus projection (rectangle in Fig. 6) **8** phallus **9** cornuti **10** female genitalia in ventral view **11** signa (rectangle in Fig. 10). Scale bar: 1 mm.

***Male genitalia*** (Figs 6–9) Uncus well-sclerotized, triangular. Tegumen with two lateral, sclerotized stripes separated by a wide membranous area. Saccus triangular. Juxta trapezoidal with a narrow, drop-like ventral projection and a wide, U-shaped dorsal projection. Labides long, narrow, setose, finger-like, distal half slightly dilated. Valva mostly membranous; costal sclerotized band not reaching apex; sacculus well-sclerotized, with a narrow, dorsal, sclerotized stripe arising from near the apex, sacculus projection narrow, strongly distally curved, with a small basal process. Phallus cylindrical, slightly longer than the costal margin of the valva; vesica with group of spine-like cornuti shorter than half of the phallus length.

***Female genitalia*** (Figs 10, 11) Papillae anales membranous, with setae. Apophyses posteriores rod-shaped, about 2.2 times length of papillae anales. Apophyses anteriores about 0.8 times the length of apophyses posteriores, with a short ventral arm near base. Lamella antevaginalis as two transverse, sclerotized stripes, not connected medially, laterally continuous with ventral arm of apophyses posteriores. Antrum membranous. Ductus bursae almost as long as antrum, sclerotized. Corpus bursae in two sections; posterior section narrow, sinuous, mainly membranous, with longitudinal folds ventrally and numerous spine-like signa arising from a dorsal sclerotized plate; anterior section membranous, spherical. Ductus seminalis a membranous projection at base of corpus bursae.

#### DNA barcodes

**Figure 12.**
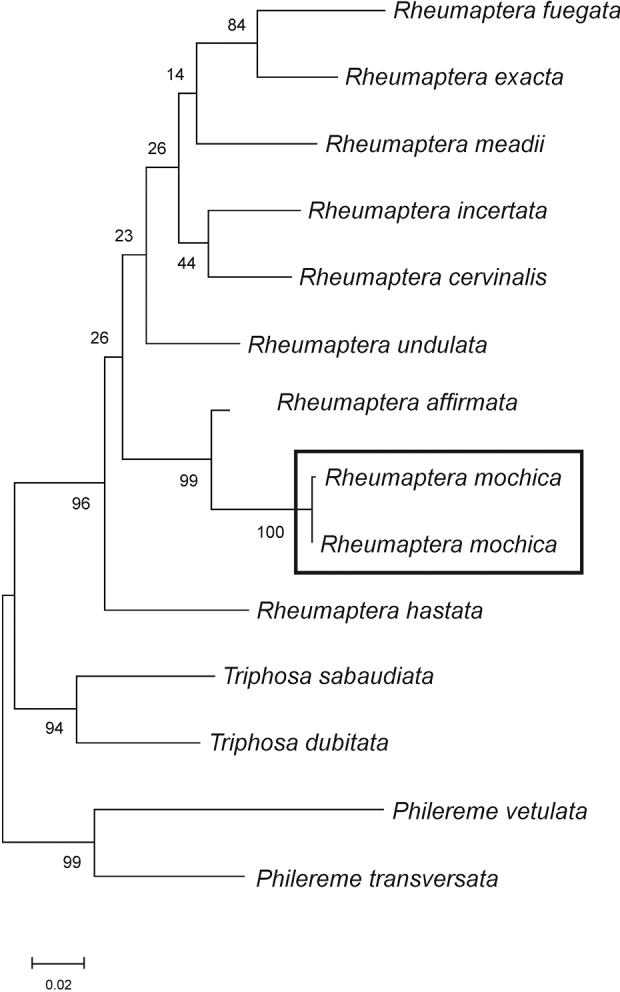
*Rheumapteramochica* (Dognin, 1904) and congeners, maximum likelihood tree of DNA barcodes. Numbers indicate bootstrap values (1000 replicates).

**(Fig. 12).** Five DNA barcodes (658 bp length) were obtained (GenBank accessions: OK484459, OK484460) from the specimens collected at Socoroma. Two haplotypes, with 0.2% (K2P) divergence between them, were detected. The sequences of *R.mochica* clustered as sister to the Neotropical congener *Rheumapteraaffirmata* (Guenée, [1858]) in the ML analysis, with 3.6–3.8% (K2P) divergence.

#### Host plant

**Figure 13.**
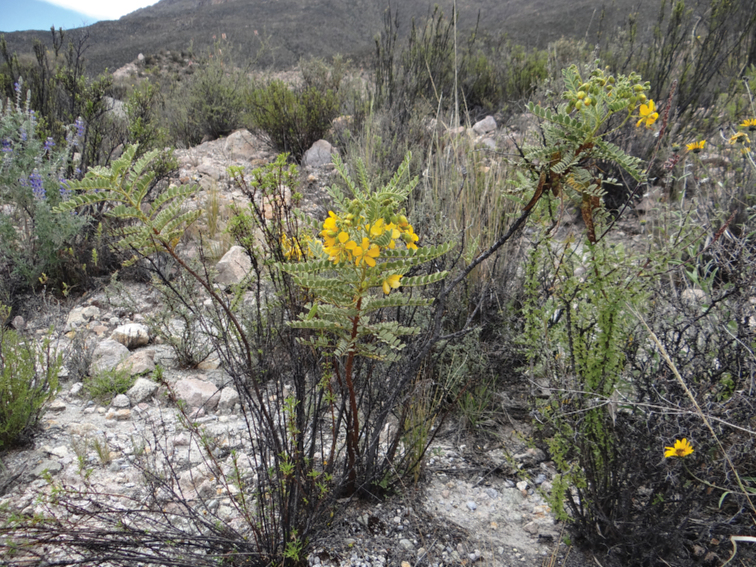
Sennabirostrisvararequipensis (Fabaceae), host plant of *R.mochica*.

**(Fig. 13).**Sennabirostrisvar.arequipensis (Fabaceae) is the first host plant recorded for *R.mochica*.

#### Geographic distribution.

**Figure 14.**
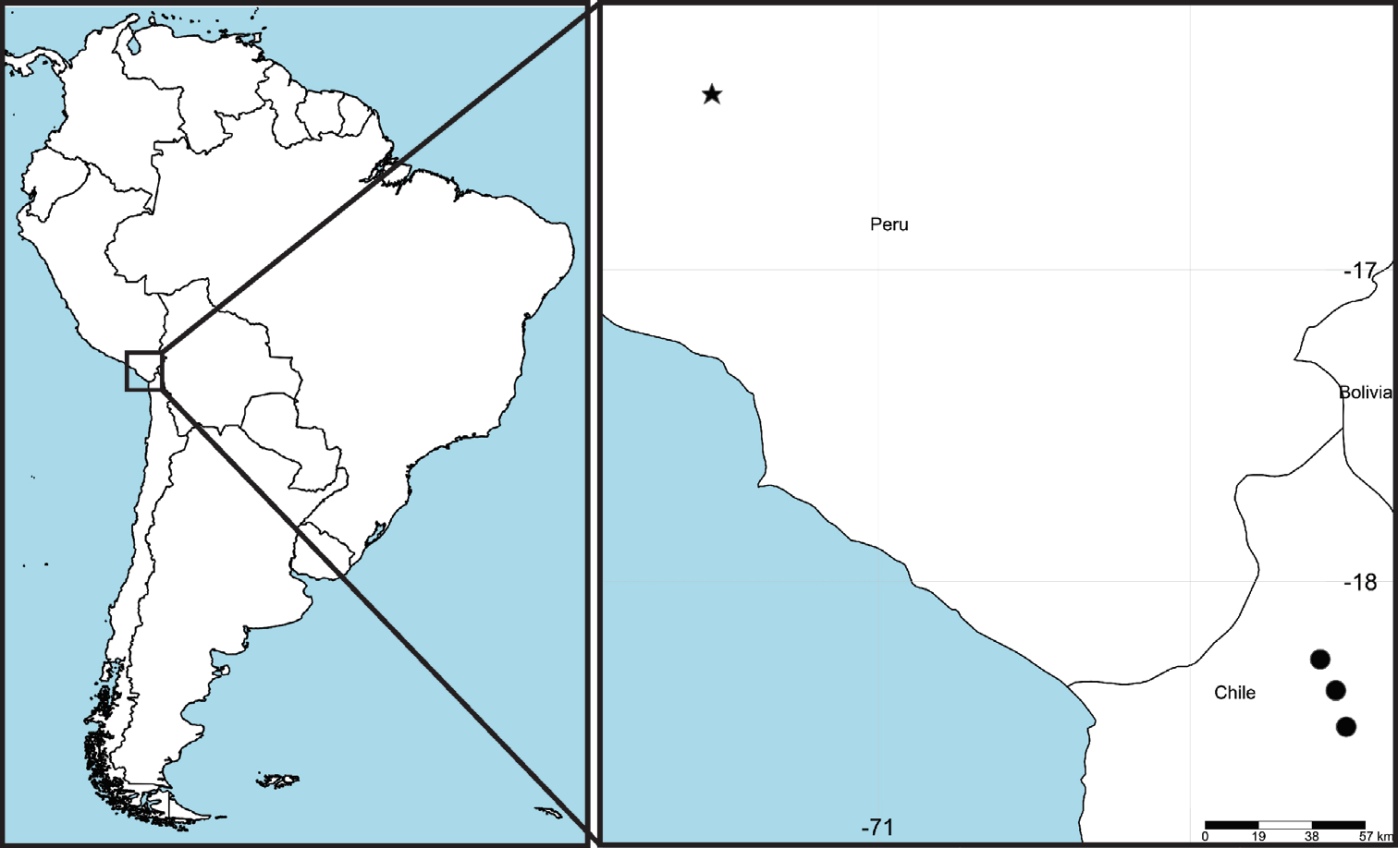
*Rheumapteramochica* (Dognin, 1904), geographic distribution. Star indicates type locality (Arequipa, Peru), circles indicate new distribution records in northern Chile.

(Fig. 14) The three localities in northern Chile represent new, expanded distribution records for *R.mochica*.

## ﻿Discussion

The moth family Geometridae is more species-rich in the Neotropical Region than in any other (Brehm et al. 2016; 2019). More than 6400 species have been described from the Neotropics (Scoble et al. 1995), many of which are known only from their type material. The specimens of *R.mochica* from northern Chile are the first to be reported in the literature after more than one hundred years since this species was described by Dognin (1904).

**Figure 15.**
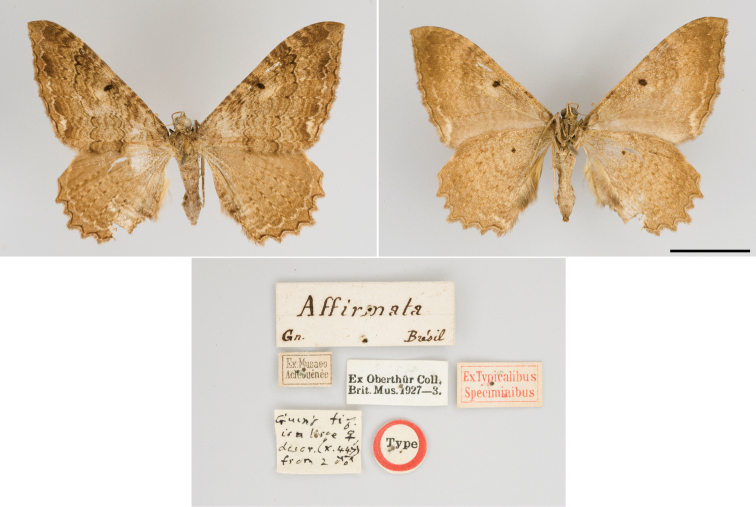
*Rheumapteraaffirmata* (Guenée, [1858]), Brazil, syntype (dorsal, ventral) and labels. Photos kindly provided by Gunnar Brehm. Scale bar: 10 mm.

The wing pattern of *R.mochica* is similar to that of the syntype of *R.affirmata* (Fig. 15). The subterminal line could be a diagnostic character to separate the two species, as this is absent or slightly differentiated in *R.mochica* (Figs 1–4), whereas this line is well-differentiated and creamy white on the fore- and hindwing of *R.affirmata*. However, additional specimens of these two Neotropical species must be examined to more accurately characterize their wing pattern, because high intraspecific variation occurs in Holarctic representatives of *Rheumaptera* (McGuffin 1973).

**Figures 16–18.**
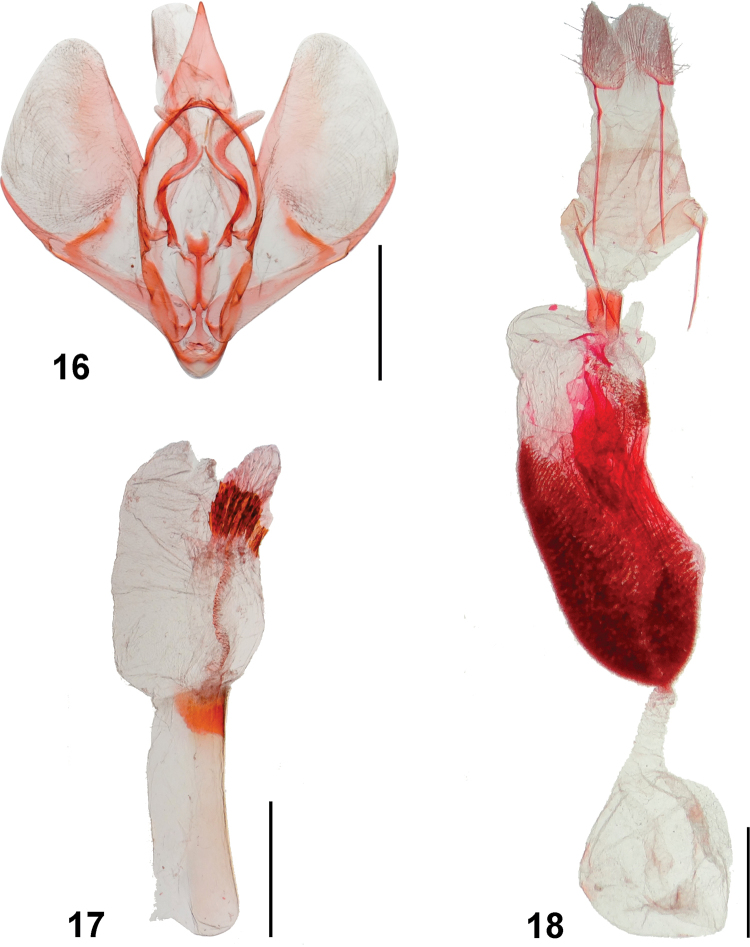
*Rheumapteraaffirmata* (Guenée, [1858]), Brazil, genitalia **16** male genitalia in ventral view, phallus removed **17** phallus **18** female genitalia, ventral view. Scale bar: 1 mm.

Genitalia morphology provides important characters for the identification of species of *Rheumaptera* and related genera (McGuffin 1973; Wanke et al. 2019). But the genitalia of *R.mochica* had remained a mystery since the species was described. The genitalia of both sexes are here described and illustrated for the first time; they are very similar to those of *R.affirmata* (Figs 16–18) based on Brazilian specimens from the DZUP collection. However, the two species can be accurately identified and separated based on morphology of the genitalia. In the male of *R.mochica*, the sacculus projection is strongly curved distally and has a small basal process, and the vesica has spine-like cornuti the longest of which is slightly shorter than half the phallus length. In contrast, the male of *R.affirmata* has the sacculus projection only slightly curved and lacks a basal process, and the vesica has serrated cornuti the longest of which is slightly shorter than a quarter of the phallus length. In the female of *R.mochica*, signa are mainly concentrated near the middle of the posterior part of the corpus bursae, whereas in *R.affirmata* signa are mainly concentrated on the anterior half of the posterior part of the corpus bursae.

This preliminary assessment of *R.mochica* provides a few interesting results, although the molecular analysis presented here was based on a single mitochondrial marker. First, *R.mochica* is confidently recovered as a member of *Rheumaptera* as proposed by Parsons et al. (1999). Second, *R.affirmata* was found to be the nearest congener to *R.mochica*, in agreement with genitalia morphology. Third, the transfer of three New World species to *Rheumaptera*, *R.affirmata*, *R.pallidividata* (Snellen, 1874), and *R.meadii* (Packard, 1874), based on a multilocus molecular analysis (Brehm et al. 2019), was supported in our analysis. Clearly, analysis of additional molecular markers and a more complete taxon sampling would provide a more robust reconstruction of the phylogenetic relationships of *R.mochica* and its congeners.

Host plants remain unknown for most species of *Rheumaptera*. Available records indicate that their host ranges can be remarkably wide, such as in the Holarctic *R.hastata* (Linnaeus, 1758) and *R.subhastata* (Nolcken, 1879), whose larvae feed on plants of at least three families (McGuffin 1973; Hausmann and Viidalepp 2012) or are restricted to a single plant genus, such as in *R.affirmata*, whose larvae feed on at least two species of *Vicia* (Fabaceae) in the Neotropics (Brehm 2002). Sennabirostrisvar.arequipensis is the first, and only, host plant ever recorded for *R.mochica*. The first author searched for geometrid larvae on other native plants in the vicinity of *Senna* sp. at the study site, including other representatives of Fabaceae (Vargas et al. 2020; Vargas 2021), but larvae of *R.mochica* were not found.

The discovery of *R.mochica* in northern Chile expands the previously documented distribution range of this geometrid moth by about 300 km to the south-east. The geographic distribution of its host plant is from southern Peru to northern Chile at elevations between 2200 and 3900 m (Irwin and Barneby 1982), encompassing the localities of the type specimens of *R.mochica* and those newly reported here.

Previous Chilean records of *Rheumaptera* were restricted to the southern zone of Chile (Parsons et al. 1999). Five species have been recorded from the rainforests of northern Patagonia at about 42°S in southern Chile (Hausmann and Parra 2009). In contrast, *R.mochica* is the first species of the genus recorded in the extremely arid environments of the northernmost part of the country. This discovery suggests that, despite their remarkable aridity, these harsh environments may harbour more undiscovered or obscure, native geometrid moths whose biology deserves further attention.

## Supplementary Material

XML Treatment for Rheumaptera mochica
